# Controlling Avian Influenza Virus in Bangladesh: Challenges and Recommendations

**DOI:** 10.3390/v12070751

**Published:** 2020-07-12

**Authors:** Rokshana Parvin, Mohammed Nooruzzaman, Congriev Kumar Kabiraj, Jahan Ara Begum, Emdadul Haque Chowdhury, Mohammad Rafiqul Islam, Timm Harder

**Affiliations:** 1 Department of Pathology, Faculty of Veterinary Science, Bangladesh Agricultural University, Mymensingh 2202, Bangladesh; rokshana.parvin@bau.edu.bd (R.P.); mohammed.nooruzzaman@bau.edu.bd (M.N.); congriev@bau.edu.bd (C.K.K.); jahan.begum@bau.edu.bd (J.A.B.); emdad001@yahoo.com (E.H.C.); mrislam_bau@yahoo.com (M.R.I.); 2 National Reference Laboratory for Avian Influenza (NRL-AI), Institute of Diagnostic Virology, Friedrich-Loeffler-Institute, Federal Research Institute for Animal Health, Suedufer 10, 17493 Greifswald-Insel Riems, Germany

**Keywords:** Bangladesh, poultry, live bird market, avian influenza virus, genotype and pathotype, control

## Abstract

Avian influenza virus (AIV) remains a huge challenge for poultry production with negative repercussions for micro- and macro-economy and public health in Bangladesh. High (HP) H5N1 and low pathogenicity (LP) H9N2 AIV are currently endemic in poultry, and both have been reported to infect humans sporadically. Multiple virus introductions of different clades of HPAIV H5N1, reassorted genotypes, and on-going diversification of LPAIV H9N2 create a highly volatile virological environment which potentially implicates increased virulence, adaptation to new host species, and subsequent zoonotic transmission. Allotropy of poultry rearing systems and supply chains further increase the risk of virus spreading, which leads to human exposure and fosters the emergence of new potentially pre-pandemic virus strains. Here, we review the epidemiology, focusing on (i) risk factors for virus spreading, (ii) viral genetic evolution, and (iii) options for AIV control in Bangladesh. It is concluded that improved control strategies would profit from the integration of various intervention tools, including effective vaccination, enhanced biosecurity practice, and improved awareness of producers and traders, although widespread household poultry rearing significantly interferes with any such strategies. Nevertheless, continuous surveillance associated with rapid diagnosis and thorough virus characterization is the basis of such strategies.

## 1. Introduction

Influenza A viruses (IAVs), belonging to the family *Orthomyxoviridae* [1], are an important cause of respiratory infections of humans and many other species of mammals and birds. Avian influenza A viruses (AIV) are potentially zoonotic pathogens that infect a wide range of avian species and occasionally spill over into mammalian species, including humans [2,3,4]. IAVs contain a negative-sense segmented RNA genome. Their eight genome segments encode for at least ten classical influenza proteins: Polymerase basic 2 (PB2), Polymerase basic 1 (PB1), Polymerase acidic (PA), Hemagglutinin (HA), Nucleoprotein (NP), Neuraminidase (NA), Matrix 1 (M1), Matrix 2 (M2), Nonstructural 1 (NS1), and Nonstructural 2 or Nuclear Export Protein (NS2/NEP), as well as, dependent on the strain, a variable number of accessory proteins (e.g., PB1-F2, PB1-N40, PA-X, PA-N182, PA-N155) through frame-shifts and by use of complementary sequences [5,6,7]. AIVs are classified into subtypes based on their surface glycoproteins, HA and NA; there are currently 16 HA and 9 NA subtypes identified in avian species. Wild aquatic birds are the natural reservoirs of AIVs [8,9]. AIVs are further categorized by their pathogenicity in chickens into high and low pathogenicity avian influenza viruses (HPAIV, LPAIV). An intravenous pathogenicity index (IVPI) in chickens is used for biological pathotype characterization by experimental infection; alternatively, the sequence of an endoproteolytic cleavage site (CS) in the HA protein (HACS) can be used as a molecular marker of pathogenicity [10]. HPAI viruses exhibit high mortality in chickens and contain a polybasic HACS, which is recognized by endogenous and ubiquitous host cellular proteases, like furin, therefore predisposing these viruses to cause systemic, often lethal, infections. In contrast, LPAI viruses invoke mild respiratory illness but also run asymptomatic courses, especially in wild bird species. The LPAIV HACS consists of a mono-, di-, or tri-basic motif which restricts proteolytic cleavage activation to extracellular trypsin-like host proteases confined to the intestinal and respiratory epithelia, respectively [11]. LPAIVs are therefore incapable of inducing systemic infection.

Aquatic birds are the natural hosts of IAV. Sporadically, viruses cross from aquatic wild birds to poultry or mammals, and new, adapted viruses may become established in these spill-over hosts. AIVs of subtypes H5 and H7 are the most important ones that cross to terrestrial poultry. These viruses have proven ability to mutate in poultry from low pathogenicity (LP) precursors circulating in wild birds into high pathogenicity (HP) viruses that multiply systemically in chickens, often causing very high mortality in infected flocks [12]. The factors governing such molecular mutation events are not fully understood. Therefore, infections of poultry with AIV of subtypes H5 and H7 are notifiable and require obligatory restriction measures [12,13]. The zoonotic A/goose/Guangdong/1/96 (gs/GD) lineage of H5N1 HPAI viruses, along with the G1 lineage of H9N2 and the Chinese H7N9 AIV, became well adapted to poultry and endemically circulate in many countries and in China, respectively [13,14,15,16]. Multiple clades/lineages and sub-lineages within these subtypes have been recognized, indicating ongoing evolution with significant genetic drift [17,18,19].

Worldwide, Bangladesh is among the countries with the highest number of reported HPAI outbreaks in poultry [20]. This is due to repeated incursions and endemic spread in poultry of HPAIV H5N1 of the gs/GD lineage since 2007 [21]. Sporadically, these HPAIVs are also detected in wild birds in Bangladesh [22,23]. LPAIV H9N2 was first detected in the country in 2006 and has likewise become endemic in poultry and is co-circulating in the country, together with HPAIV [24,25]. Further AIV subtypes were isolated intermittently from domestic free-range birds and, more rarely, from aquatic wild birds [22,23,26]. The widespread continuous co-circulation of HPAIV H5N1 and LPAIV H9N2 bears increased risks for the potential generation of new sub- and genotypes of AIVs which constitute additional obstacles to virus eradication. Both viruses cause significant economic damage in poultry production and threaten public health by their zoonotic propensity [27]. AIV surveillance studies in Bangladesh [22,26,28,29] have shown that domestic ducks play an important role in the transmission and emergence of new AIV sub- and genotypes. 

In this review, we focus on specific poultry rearing and trading habits in the country and examine their potential impact as risk factors for virus spread and zoonotic transmission. Preventive strategies are proposed to control and eradicate the disease in this specific framework.

## 2. Ecology and Epidemiology of AIVs in Bangladesh

### 2.1. Geographical and Ecological Frameworks

**Figure 1:**
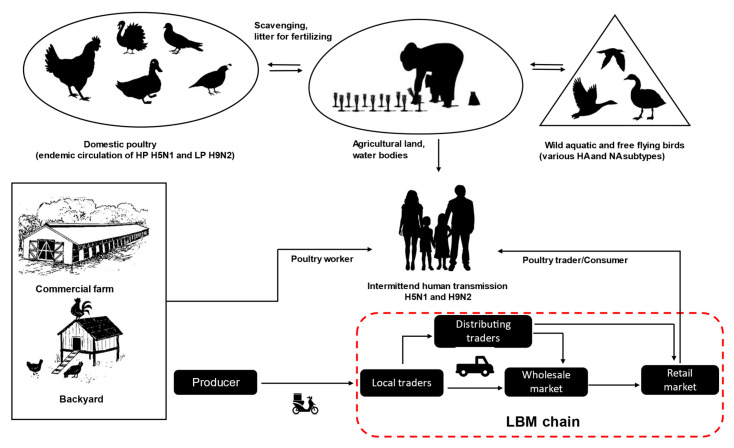
An overview of avian influenza virus transmission and live bird market trading chains (red dashed line) in Bangladesh.

Bangladesh is a low to middle-income country in South Asia agriculturally characterized by rich water environments, paddy rice farming, and poultry production. The economy is heavily dependent on agriculture and livestock production. The country consists of a broad, deltaic plain with many tributaries and a sea coast with an extensive mangrove belt. It is at high risk of frequent flooding by three major rivers, the Ganges, Jamuna, and Brahmaputra. In addition, there is annual flooding from the seaside due to cyclones in the Bengal Bay of the Indian Ocean. Bangladesh is also an attractive and important wintering site for wild migratory birds, in particular, of the order *Anseriformes*, which breed in arctic and palearctic regions of Russia. Moreover, two major migratory bird flyways, the Central Asian and East Asian-Australian, are crossing Bangladesh [30,31,32]. The abundance of shallow coastal wetlands and vast inland wetlands (so-called haors) provide a large reservoir for wildlife, especially waterfowl, which migrate from many parts of Russia and Central Asia during winter [22,33]. This creates an ecological scenario where wild aquatic birds, domestic ducks and galliform poultry intermingle and in which pathogens, like AIV, can be easily exchanged (Figure 1). According to farmers’ complains, the outbreaks are more common during the autumn and winter; however, studies were unable to identify any distinct seasonality for endemic H5N1 and H9N2 virus outbreaks in Bangladesh, and AIVs have been frequently identified from poultry in live bird markets (LBM) throughout the year [34,35,36,37]. This indicates that endemic virus circulation in poultry populations is likely the most important driver in this scenario.

### 2.2. Poultry Rearing Systems and Trading Chains

Poultry rearing in Bangladesh comprises commercial and backyard poultry production. Commercial farming can be further categorized according to farm sizes following the sectoring approach of the Food and Agriculture Organization of the United States (FAO) [38]: large-scale breeder farming, medium size farming (broiler, layer, and duck), and small size farming (layer, broiler, quail, pigeon, turkey, guinea fowl, etc.). In Bangladesh, more than 60% of the people live in rural villages [39], and it is estimated that about 90% of the rural households raise poultry (chicken, duck, pigeon) in traditional backyard settings [40]. The main poultry types reared and traded in the country include: Industrial white-feathered broiler chickens [38], Sonali (cross-breed of Rhode Island Red cocks and Fayoumi hens) [41], Deshi (back-yard chickens) [42], and ducks. Broiler, Sonali, and few ducks are raised in commercial settings, while Deshi and most of the ducks are reared in traditional scavenging systems [43,44]. Many further commercial poultry breeds, such as hybrid layer, have successfully been established in recent years in Bangladesh and are being profitably utilized by different entrepreneurs [45]. 

At the beginning of 21st century, the Bangladeshi poultry industry expanded rapidly. The Government of Bangladesh has given top priority to livestock development to meet the growing demand for high quality animal protein in the human diet and to create employment opportunities and generate income for the low-income rural population. As such, both industrial poultry production and family poultry rearing are supported. Bangladesh currently raises an estimated 282 million of chickens and 55 million of ducks [46]. Improper management and biosecurity practices in poultry rearing have fostered the emergence and re-emergence of economically important infectious disease, like HPAI, leading to endemic spread of HPAIV H5N1 and LPAIV H9N2 [27,47]. During the first and second wave of HPAI H5N1 in 2007 and 2008, approximately 547 poultry farms had been affected that forced the authority to cull nearly 1.7 million birds, resulting in substantial financial losses [48]. Against the general market rules where shortage of products induces rising prices, the price of poultry meat and eggs in Bangladesh declined by 27% as a majority of the consumers desisted from consumption of potentially unsafe broiler meat and chicken eggs [48]. Finally, the poultry market collapsed, and many farm owners lost all capital. 

LBMs have been incriminated in the dynamics of AIVs transmission, dissemination, and persistent circulation, thus facilitating the reassortment between different virus strains in many countries [49,50]. In LBMs, mixing of different species of birds (chickens, ducks, geese, pigeons, etc.) from different sources (wild birds, backyards, and commercial farms) creates a suitable niche for persistence and perpetuation of AIVs. In Bangladesh, live bird trading is ubiquitous, and LBMs distribute 95% of the total poultry meat and egg retails [38]. Birds are mostly traded alive because of cultural and religious preferences for consuming freshly slaughtered poultry. There is an apparent lack of processed meat marketing facilities and cold chains, particularly among rural households [51]. LBMs in rural Bangladesh often do not provide even a minimum level of biosecurity and lack proper disinfection and sanitation procedures. Before finally sold in LBMs, birds often move through a complex trading network of peripheral rural markets via several transshipment stations to wholesalers in city markets, thereby increasing risks for AIV spread and transmission to humans (Figure 1). Since 2008, several subtypes of AIVs, including, predominantly, HPAIV H5N1 and LPAIV H9N2, have been isolated from LBMs in Bangladesh [34,35,36,50]. Unhygienic slaughtering processes at LBMs bear increased risks of zoonotic transmissions [34,50], but actual virus transmission to LBM workers as evidenced by seroconversion was scarce [52]. Yet, LBMs remain a very important target to understand and intercept the local circulation of AIVs in domestic poultry in Bangladesh and to identify and combat risk factors in zoonotic transmission. The outbreak frequency of endemic HPAI H5N1 and LPAI H9N2 has increased due to increased poultry production, characterized by a mélange of various, highly fragmented rearing systems and marketing chains [53], as elucidated above.

### 2.3. Virus Transmission and Risk Factors

Droplet, aerosol, faeco-oral, and direct or indirect contact with contaminated materials are widely described modes of transmission of AIVs [54]. Transmission risks increase depending on host susceptibility and viral load in the environment, as well as on distance between and frequency of contacts [55]. In Bangladesh, the domestic duck is currently considered as the most important epidemiological factor associated with AIV transmission between wild birds and other poultry [22,56]. Low awareness among the raisers of backyard poultry of the zoonotic properties of AIV, neglected practice of biosecurity measures, and the close living arrangements of poultry and rural human populations lodge them at the highest risk for zoonotic transmission [27]. HPAI H5N1 and LPAI H9N2 outbreaks cause enormous losses to small scale poultry producers [57]. Safe disposal of litter and fallen animals pose further severe problems. Untreated poultry litters are being used as fertilizer on agricultural lands and as fish feed in water bodies, which may contaminate the environment and further trigger viral spreading [58]. However, large-scale commercial farms are relatively better managed and follow biosecurity recommendations, which reduces huge financial losses due to AI outbreaks [27]. Moreover, LBMs provide foraging opportunities for peri-domestic birds, such as crows, sparrows, and starlings [59]. Consequently, house crows have been found positive for HPAIV H5N1 on several occasions [60]. Moreover, no closure days for LBMs with H5 AIV-positive birds are usually decreed. Possible risk factors stratified according to poultry rearing and marketing systems in Bangladesh are listed in Table 1. Effective human-human transmission of HPAI or LPAI virus is not evident in the country, but avian–human transmission follows the close proximity of each other [27,50].

## 3. Overview of AIV Sub- and Pathotypes in Bangladesh

**Figure 2:**
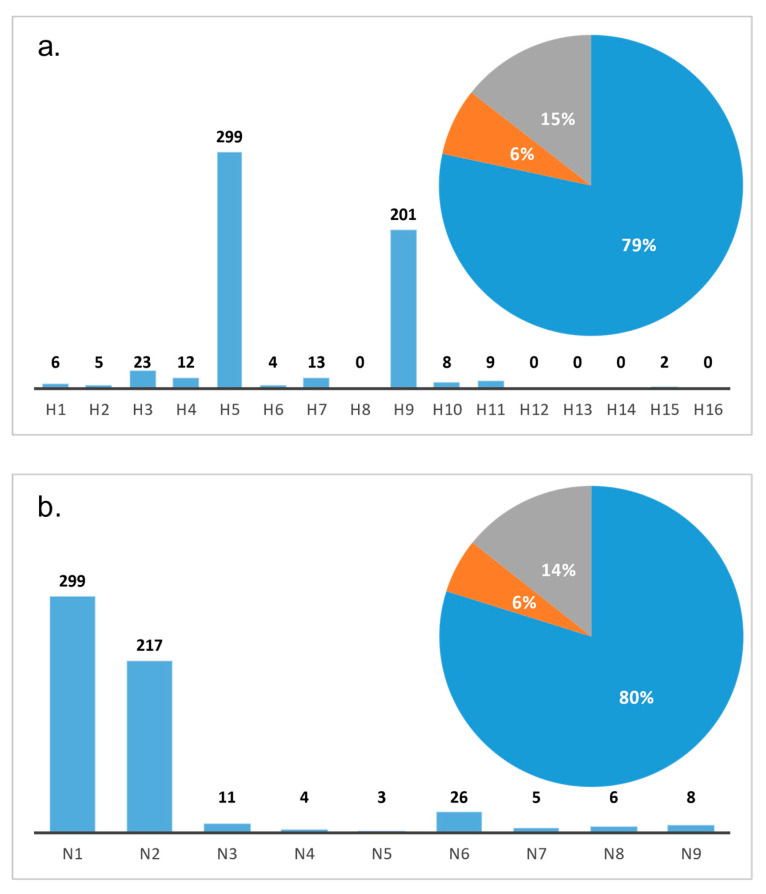
Occurrence of avian influenza virus subtypes in Bangladesh based on hemagglutinin (*HA*) (**a**) and neuraminidase (*NA*) (**b**) sequences available in public databases and distribution of host species origin (blue—poultry (79% and 80%), orange—wild birds (6% each), and grey—environment (15% and 14%)).

Viral nucleotide sequences of AIVs detected in Bangladesh were retrieved from public sequence databases (GenBank at National Center for Biotechnology Information (NCBI) or EpiFlu of the Global Initiative on Sharing Avian Influenza Data (GISAID) or Influenza Research Database (IRD)). Until December 2019, a total of 582 fully sequenced avian origin AIV isolates were available. About 79% of these were obtained from domestic birds (*n* = 462 *HA*, 461 *NA*), 14% from environmental samples (*n* = 84 *HA*, 83 *NA*) and 6% from wild birds (*n* = 36 *HA*, 35 *NA*) (Figure 2a,b, Supplemental Table S1 and Table S2). Although a wide variation of different subtypes was described, HPAIV H5N1 and LPAIV H9N2 constitute the dominant circulating subtypes in poultry (Figure 2a,b) and are endemic in the country [47]. 

The majority of frequently identified AIV subtypes arise from outbreaks in backyard, small to medium commercial farms and from surveillance sampling at LBMs [30,56,73,74,75]. Sampling from wild aquatic birds appears to be more unsteady [22,36,76]. Restricting this analysis to sequenced viruses may introduce a bias; however, all surveillance data, including unsequenced samples and isolates that are available from LBMs, environmental, and outbreak sampling, confirm a grossly higher detection rate for H5N1 and H9N2 subtypes compared to any other subtype [22,37,56,62,64].

### 3.1. HPAI Viruses

In the 21st century, HPAI viruses eventfully affected domestic poultry with significant public health and economic concerns throughout South East Asia. In particular, the H5N1 HPAI viruses of the gs/GD lineage, soon after its emergence in Guangdong, China [77], have become endemically established in domestic poultry across Asia and Africa or have caused excessive epidemics throughout Europe and in North America [78]. The inherent high mutation rate of influenza A viruses along with frequent reassortments of gene segments with different AIVs led to the emergence of numerous distinct phylogenetic clades of the H5 *HA* and various sub- and genotypes of the original HPAIV H5N1 strain [79]. As a fact, the *HA* gene segment of the gs/GD lineage has segregated into 40 different clades during the past 20 years and is still evolving [17]. 

**Figure 3:**
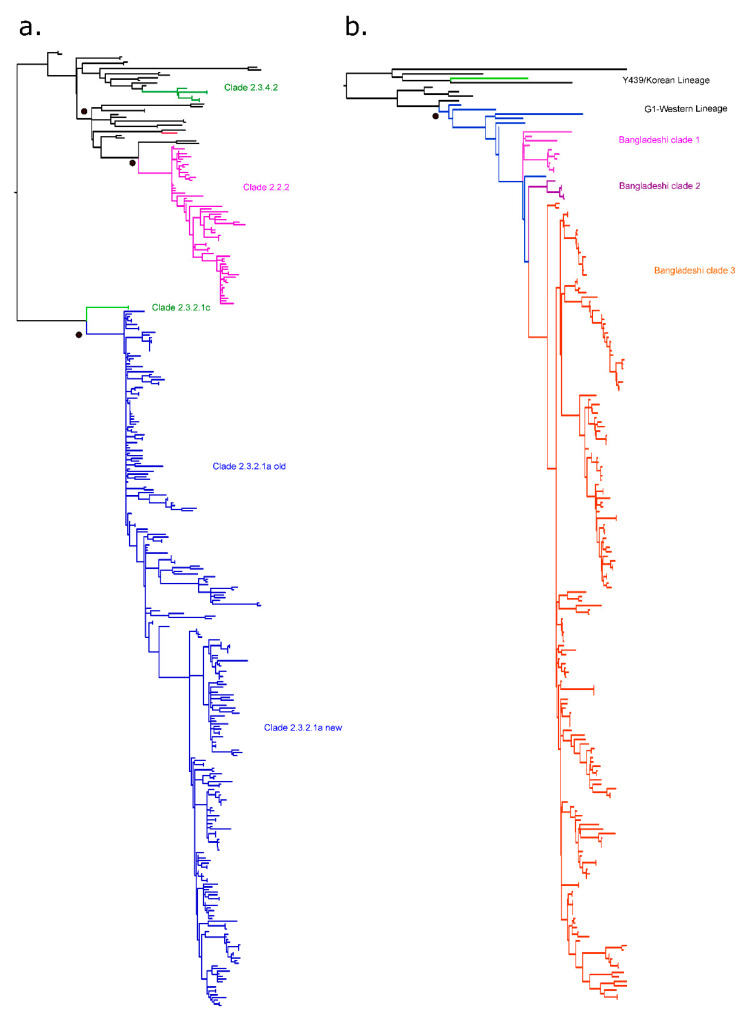
Phylogenetic relationship of *HA* genes of Bangladeshi high pathogenicity (HP) H5N1 (**a**) and low pathogenicity (LP) H9N2 (**b**) viruses available in public databases (282 H5N1 *HA* and 201 H9N2 *HA*). The trees were generated by use of a maximum likelihood tree method and ultrafast bootstrapping with 1000 replicates with the IQ-Tree software [97,98]. ModelFinder embedded in IQ-Tree [99] was used to select the best fitted substitution model according to the Bayesian information criterion (GTR+F+I+G4). The colored branches represent isolates detected in Bangladesh. Black circles indicate the approximate position in each clade of the vaccine strains used or licensed for use in Bangladesh. Extended versions of these trees are shown in Supplemental Figure S1.

In Bangladesh, the first outbreak of HPAI of H5N1 subtype in poultry was recorded in 2007 [21]. As of 20 May 2020, a total of 561 outbreaks had been reported to the World Organization for Animal Health (Office International des Epizooties, OIE) involving viruses of five distinct gs/GD clades: 2.2.2, 2.3.2.1a, 2.3.2.1c, 2.3.4.2, and 2.3.4.4b (Figure 3a and Supplemental Figure S1a) [80]. The initial outbreaks are linked to viruses of clade 2.2.2, which were closely related to isolates from Mongolia and Russia [81], suggestive of an incursion route to Bangladesh through migratory birds [21,81]. H5N1 viruses of clade 2.2.2 were responsible for the majority of the outbreaks in Bangladesh between 2007 and 2011. Two human infections with clade 2.2.2 were also detected in 2011 [82]. Later in 2011, two new clades, 2.3.4.2 and 2.3.2.1 (later designated as clade 2.3.2.1a), were introduced into the country [60,73,75,83]. Migratory birds and cross-border poultry trading were suspected to have played a major role in those introductions [30,84]. Viruses of clade 2.3.4.2 only circulated for a very brief period in 2011 and disappeared rapidly. Similarly, viruses of clade 2.3.2.1c prevailed for a very brief period in 2012 and became extinct thereafter. Interestingly, the latter two clades, 2.3.4.2 and 2.3.2.1c, have been detected in chickens at LBM but never in wild birds [85]. Since 2014, only viruses of the clade 2.3.2.1a prevailed in domestic poultry, wild and migratory birds, and in LBMs of Bangladesh [30,47,70,74,75]. The only fatal human HPAIV infection was detected in Bangladesh in 2013 and was caused by a clade 2.3.2.1a virus [86]. Rapid accumulation of point mutations and reassortments shaped the subsequent evolution of clade 2.3.2.1a viruses in Bangladesh. Some isolates harbored a *PB1* gene segment of the co-circulating H9N2 LPAI viruses [75,87]; such viruses were detected between 2011 and 2015 and disappeared thereafter (Table 2). In June 2015, a new reassortant genotype of the clade 2.3.2.1a (here designated as clade 2.3.2.1a (new) R1) was detected in apparently healthy free-range ducks in wetland areas and poultry in LBMs [22]. The reassortment events shaping this genotype started even earlier as four surveillance samples from domestic ducks obtained in 2013 had a similar gene constellation [74]. These viruses are reassortants between HPAI and LPAI viruses: Their *HA*, *NA*, and *M* genes derived from circulating clade 2.3.2.1a viruses, whereas *PB2*, *PB1*, *PA*, *NP*, and *NS* genes originated from unknown Eurasian LPAI viruses (Table 3). Of note, the *HA* gene of the new clade 2.3.2.1a viruses is about 2.7% divergent from the old clade 2.3.2.1a viruses, but, in only two out of 66 isolates examined, amino acid substitutions in antigenic sites 1 (position 141) and 3 (position 129), respectively, were noticed [74]. Subsequently, this genotype has been extensively detected in domestic poultry associated with clinical outbreaks [74]. In addition, these viruses further reassorted and gained a new *PA* gene segment, thereby evolving to clade 2.3.2.1a (new) R2. These viruses have been detected during surveillance in LBMs since May 2017 [76]. The *PA* gene of the reassortant clade 2.3.2.1a (new) R2 showed close similarity with Bangladeshi H3N8-like LPAI viruses detected in domestic ducks (Table 3). Currently both R1 and R2 reassortant genotypes of H5N1 HPAIV co-circulate in Bangladesh.

An overview of the complex evolution of the gene constellation of 220 Bangladeshi H5N1 HPAI viruses based on their complete genome sequences available in the GISAID and NCBI databases is provided in Table 3. A total of 12 H5N1 viruses clustering with the clade 2.2.2 were detected during 2007 and 2011. Two isolates of each clade 2.3.4.2 and clade 2.3.2.1c were reported in 2011 and 2012, respectively. Of note, the *M* gene of the two 2.3.2.1c H5N1 viruses clustered with the Chinese H9N2-like viruses [74]. A total of 84 H5N1 viruses have been detected from clade 2.3.2.1a without any reassortment event, and 8 H5N1 viruses of clade 2.3.2.1a have carried the *PB1* gene derived from Bangladeshi H9N2 AIVs identified between 2011 and 2015. Viruses of this genotype were no longer detected after 2015. Viruses of the clade 2.3.2.1a (new) R1 (*n* = 98) and clade 2.3.2.1a (new) R2 (*n* = 14) have been circulating since 2015 and 2017, respectively. More recently, Bangladesh reported another novel HPAI incursion: HPAIV H5N6 has been detected in chickens and waterfowl through active LBM surveillance during 2016–2017 [23,93]. The newly emerged HPAI H5N6 viruses belong to the clade 2.3.4.4b and are likely introduced into Bangladesh via migratory birds during the winter season of 2015–2016. Human infections with influenza clade 2.3.4.4 viruses in Bangladesh have not been identified, but the viruses have several molecular markers associated with potential human infection [23].

LPAI viruses are found worldwide in aquatic wild birds which constitute the main reservoir. Poultry can be infected by many of those subtypes, among which H9N2 LPAIVs are the most significant and have become endemic in poultry in many countries of Asia, the Middle East, and Africa. Although currently neglected by international control legislations, H9N2 viruses play a major role as an avian pathogen leading to considerable production loss. In addition, these viruses contributed massively to genetic reassortment events, thereby shaping the vast genetic variability of gs/GD HPAIV [55]. The first reported introduction of H9N2 viruses into Bangladesh occurred in 2006 in a commercial breeder [24]. Infected flocks suffered from a significant drop in egg production and hatchability and reported up to 50% morbidity and 3% mortality [24]. Phylogenetically, the causative virus was identified as a member of the so-called “western” G1 lineage of H9N2 [55]. The virus is believed to be introduced by legal or illegal regional trade of live poultry, pet, or imported feed components from the neighboring countries [25,35]. Since the first outbreak, H9N2 viruses of the G1 lineage are being frequently identified in commercial and backyard poultry and in surveillance samples from LBMs, indicating endemic virus circulation in poultry [35,36,94]. A single virus from an environmental sample of domestic ducks identified in 2009 clustered with the Y439 lineage (also known as Korean or Eurasian wild bird lineage) of the H9N2 subtype, but apparently this lineage did not become established [35,55]. The H9 *HA* phylogeny (HPAI—Figure 3b, Supplemental Figure S1b) showed that, since 2010, Bangladeshi H9N2 viruses further segregated into three major clades here designated as Bangladeshi clade 1–3 all rooted in the “western” clade of the G1 lineage (Figure 3b). The Bangladeshi clade 3 formed further divergent subclusters. The predominant G1-lineage of these viruses appears to have originated from a reassortment event between co-circulating HPAIV H7N3 and LPAIV H9N2 viruses in Pakistan [25,35,95], which were introduced to and evolved over time in Bangladesh [34,94]. Moreover, the LPAI H9N2 viruses circulating in Bangladesh have shown to accumulate molecular markers that favor interspecies transmission [25,35], eventually resulting in sporadic human transmission in Bangladesh (Table 2). The Bangladeshi H9N2 viruses initially expressed a di-basic (PAKSSR*GLF) cleavage site in the HA protein [25]. Later strains showed expression of a tri-basic (PAKSKR*GLF) HA cleavage site motif [25,35]; the potential pathogenic impact of the tri-basic HACS has been recently examined but could not be unambiguously related with increased pathogenicity in chickens [25,35,96]. 

### 3.2. LPAI Viruses

Despite prolonged co-circulation, reassortment events between HPAI H5N1 of clade 2.3.2.1a (old) and LPAI H9N2 viruses had been grossly limited [22,74,87]. It is unclear whether a genetic incompatibility existed between these two circulating virus subtypes that could have resulted in a lack of frequent reassortments. In contrast, the newly emerging H5N1 viruses of the clade 2.3.2.1a (new) that have quickly become predominant contained internal genes from non-H9N2 LPAI viruses [22,74]. Surprisingly, a recent study has reported co-subsistence of 5 different subtypes of HPAI and LPAI with 8 reassortant genotypes of AIVs after plaque purification in clinical samples from commercial chickens and domestic ducks in Bangladesh [56]. The unexpected findings of mixtures of HPAIV H5, H7 and LPAIV H9N2 further complicate the diagnosis and disease control in Bangladesh. Because of the virus potential for generating novel reassortant variants between similar or with other virus subtypes, their persistent circulation in poultry is a serious threat to animal and human health.

Apart from the endemic LPAI H9N2 viruses, other LPAI virus subtypes identified in domestic birds from LBM samples in Bangladesh include H4N6 and H11N3. Subtypes H1N1, H1N3, H1N5, H2N4, H2N5, H3N1, H3N2, H4N2, H5N2, H6N1, H7N1, H7N5, and H7N9 (Supplemental Table S1) have been detected sporadically and mostly in domestic birds, especially backyard ducks or ducks sold in LBMs, but seldom in wild birds [22,29,36]. 

### 3.3. Zoonotic Transmission

AIVs, in particular viruses of the HPAI H5N1, LPAI H9N2 G1, and HPAI/LPAI H7N9 lineages, occasionally cross the species barrier from birds to mammals, including humans [100,101,102,103]. Among these subtypes H5N1 and H7N9 are of great public health concern due to 861 and 1625 laboratory-confirmed human cases causing 455 and 623 deaths, respectively, globally reported to the World Health Organization [104,105]. Bangladesh has so far confirmed 11 human AIV infections, 8 with HPAIV H5N1 with a single fatal case and 3 non-fatal LPAIV H9N2 infections (Table 2). Close contact to contaminated LBMs and infected backyard birds have been claimed to be the potential source of human infections with H5N1 and H9N2 in Bangladesh where both adults and children were affected (references listed in Table 2). Human infections with zoonotic influenza viruses are grossly less frequent than human seasonal influenza cases; however, human AIV infections may lead to virus adaptation and the emergence of potentially new pandemic viruses [106].

## 4. Towards Effective Control and Prevention

Eradication of the economically important AIVs in Bangladesh is so far unsuccessful, and outbreaks are still seen in all sectors of poultry production [27]. During early outbreaks ‘stamping out’ culling of potentially infected birds has been used as a front line of control in many countries, including Bangladesh [107,108]. For countries like Bangladesh, while facing endemic infections and limited financial sources for farmers’ compensation, a rigorous stamping out policy has become uneconomical and unfeasible. Current practices in Bangladesh include the isolation of HPAIV-infected flocks having relatively lower mortality and stamping out when mortality is higher. Official reporting to the Department of Livestock Services (DLS) is practiced in the latter case.

Many countries, including China, Indonesia, Egypt, South Korea, Morocco, Iran, Pakistan, and United Arab Emirates, have espoused vaccination as a key approach to combat AIV outbreaks in poultry, especially when facing entrenchment of potentially zoonotic viruses in poultry populations [49,107,109,110,111,112]. Apart from clinical protection of poultry, vaccination aims at reducing the shedding of viruses and, thereby, at decreasing the amount of virus in the environment and at the poultry-human interface [113,114]. On the other side, vaccination-built population immunity may impose a selection pressure and enforces viral antigenic drift. Drift variants would escape vaccination-induced immunity and continue to spread. Therefore, effective vaccination programs require close-meshed supervision, which adds to the costs of purchasing, distributing, and applying the vaccines [97,98]. Therefore, the Government of Bangladesh (GoB) decided in 2012 to embark on avian influenza vaccination. Initially three vaccines, Vectormune HVT-AI (Ceva), inactivated Re-6 (Merial), and inactivated Nobilis H5 (MSD), were introduced on an experimental basis in two districts (Gazipur and Kishoreganj). These vaccines are based on virus isolates A/swan/Hungary/4999/2006, HP H5N1, clade 2.2 (Ceva), A/dk/GD/S1322/2010, HP H5N1, clade 2.3.2.1 (Merial), and A/duck/Potsdam/1402/1986, LP H5N2 (MSD). Vectormune HVT-AI is to be administered as a single shot to day old chicks at the hatchery, while Re-6 or Nobilis H5 are for vaccination of pullets of any age and require two doses at 6–8 weeks interval. Vaccination coverage in the two districts was 100% for parent stocks but only 32% and 54% in commercial layer flocks in Kishoreganj and Gazipur districts, respectively, during the study period of 8 months from December 2012 to August 2013. An expert committee was formed to evaluate the vaccination field trial. Department of Livestock Services (DLS), GoB, monitored the field trial and evaluated the serological response in collaboration with Bangladesh Livestock Research Institute (BLRI), Central Disease Investigation Laboratory (CDIL) and Bangladesh Agricultural University (Pathology and Microbiology departments). After 12 weeks post-vaccination, HI antibody titers, measured against a local isolate of H5N1 (clade 2.3.2.1a), ranging above log_2_ 5 was found in 83% of the vaccinated flocks that had received either HVT-AI or Re-6 and 47% with Nobilis H5 (unpublished, personal communication, M. R. Islam). From 2014 onward, all the three vaccines were permitted by the GoB for commercial use across the country; however, only HVT-AI and Re-6 vaccines continue to be marketed [115,116]. However, since then, there has been no regular monitoring of the vaccination program, and vaccines are now being used sporadically at farmers’ will. The spread of HPAIV H5N1 apparently has not been disrupted as the virus is continuously detected at LBMs, as well as in the field [22,36,56,74]. In addition, antigenic drift in association with vaccination has been reported from other countries; however, the—possibly—low coverage of vaccination in Bangladesh is not expected to have a significant impact on antigenic selection pressure [49]. Vaccination against LPAI H9N2 viruses using another inactivated CEVAC FLU H9 K (Ceva, a G1-like Middle East H9N2 isolate) has recently been approved as a first H9-specific vaccine in Bangladesh by the DLS following a serological trial (data unavailable), and the vaccine is already available in the market. Yet, the whole backyard sector and a vast majority of medium to small size farms, as well as domestic ducks, remain unvaccinated.

While there are scopes for making the vaccination program more effective through monitoring and regulatory measures, the most important tool for better disease control is the improvement of farm management and biosecurity practices in all poultry operations and in LBMs. A general lack of awareness and limited biosecurity practice by poultry raisers and traders in LBMs is significantly associated with a higher likelihood to detect AIVs in the country [43,50,59,72,117]. To improve the situation, GoB, supported by international agencies, such as FAO, has taken a number of initiatives, including nationwide public awareness-raising campaigns and adoption of basic biosecurity measures at farm level [27]. Despite many efforts, the situation has not yet improved much. In Bangladesh, biosecurity measures are at an agreeable level only in large breeder commercial farms. Consequently, current and future efforts to rise levels of biosecurity practices must focus on small to medium scale poultry farms and should include stakeholders in the supply chains. Backyard poultry constitutes yet a different but leading problem. The Department of Pathology, Bangladesh Agricultural University (BAU), Mymensingh, recently assessed the biosecurity status in small and medium scale layer farms of two selected areas through a community participatory approach. Based on the risks identified and farmers’ do-able capacity, a “10 + 10 biosecurity model”, comprising 10 conceptual and structural and 10 operational guidelines, was proposed for the improvement of farm biosecurity. The model was disseminated to the farmers through a community approach. Adoption of the suggested measures by the farmers had a positive impact and reduced poultry mortality due to disease outbreaks and treatment costs (information received from project document of M. R. Islam, manuscript in preparation). The model was implanted in selected intervention farms, and evaluations regarding the reduction of disease outbreaks and treatment costs are continuing.

The following general guidelines may further help in the prevention and control of avian influenza in poultry farms in Bangladesh: Each and every farm needs to be considered separately. The farms have to be analyzed for the biosecurity risks using a standardized risk analysis framework that includes risk assessment, management, and communication. Once the risks are identified, management options may be defined, some of which have to be communicated and implemented at the farm level, and some of which target the community or even the national level. Problems of analyses and implementation are usually reciprocally related to the size of the holding putting the household level and backyard poultry at the biggest challenge. The community-based approach could be a good option in such situation. Simultaneously, the governmental biosecurity guidelines for poultry farming and their compliance must be enforced and monitored by the regulatory authority at all levels of the poultry supply chain. Collectively, the following interventions may further improve such control plans:

1. Improving biosecurity:
  - Biosecurity and compliance levels must be harmonized, monitored, and enforced.
  - Training and awareness for all level of stakeholders, including managers, farmers, and traders; training courses on biosecurity, rapid outbreak response, communication programs.
  - Regulations for small scale commercial and backyard poultry under avian influenza vaccination, including community engagement in biosecurity programs, e.g., households have to be encouraged to keep backyard chickens and ducks in separate night shelters.
2. Tightening of trading control and biosecurity at LBMs:
  - Reduce inter-district/inter-regional poultry trading through promotion of regional trading.
  - Avoid mixing of birds from different sources and days. Birds of different species have to be kept separately and isolated.
  - Enhanced disease control at the sources, including upgrading poultry wholesale markets and slaughterhouses and further processing of poultry and selling in the supermarkets, which may help in the gradual phasing out of LBMs.
  - Encourage to wear personal protective equipment when working at LBMs, regular cleaning and disinfection (C&D) of the market area, consider decreeing market closure days once per week for thorough C&D.
  - Avoid mixing of wild migratory birds and domestic ducks during winter season and spring.
3. General:
  - Monitoring both H5N1 and H9N2 vaccination across the country by the government regulatory authority through their field service network.
  - Updating response policy to H5N1 outbreaks in the context of vaccination programs.
  - Increasing medium- and long-term capacities of the veterinary and public health systems to strengthen the emergency response to disease outbreaks.
  - Promoting the “One Health” approach to ensure cross sector coordination in control of AI outbreaks.

## 5. Conclusions

Co-circulation of zoonotic and endemic HPAI H5N1 and LPAI H9N2 viruses in poultry, the recent emergence of a new strain of HPAIV (H5N6) in domestic birds, and the sporadic identification of various other AIV subtypes in poultry and wild bird populations have created a complicated epidemiological situation in Bangladesh. The Bangladeshi H5N1 and H9N2 viruses have evolved into various divergent clusters. Although there currently seems to be a declining trend of HPAI H5N1, this may well be due to a lack of actual disease reporting at farm level and requires virologically convincing evidence. Expansive circulation of non-notifiable H9N2 throughout the whole country currently bears great economic damage to poultry production. Intensified surveillance is at the basis of all efforts to improve AIV-targeted disease control in the country. Public education towards improved awareness and biosecurity is a lengthy process and not expected to yield rapid success. Cost-effective and sensible implementation of avian influenza vaccinations would greatly benefit from well-planned concerted actions of GoB and stakeholders and also does essentially involve collateral virological and serological monitoring of vaccinated flocks. Geographical constraints and the dynamics of the human population in Bangladesh pose severe obstacles to the implementation of improved control measures. 

## Figures and Tables

**Table 1:** Risk factors identified in the Bangladeshi poultry farming and marketing systems that may promote spread of avian influenza virus (AIV).

Sectors	Possible Risk Factors	References
**Backyard poultry**	Frequent close contact with neighboring poultry flocks due to free scavenging Co-rearing of different species (chicken, duck, parrot, pigeon) Handling and slaughtering of sick birds, including putting sick poultry together Uncontrolled movement and traffic Lack of regular cleaning, safe disposal and disinfection, including feeding of slaughter remnants of purchased chickens to backyard chickens Ubiquitous water bodies, rice paddies, and bushland fostering contact with wild bird populations	[27,40,43,61,62,63,64,65,66,67]
**Commercial poultry**		
**Small and medium enterprises**	Farm location near public places and high density of farms Lack of isolation facilities and improper disposal of sick or dead birds Lack of biosecurity (fencing and improper using of footbaths) leading to contact with free flying wild and backyard birds and other animals Exchange of farm equipment Uncontrolled vehicle movement Ubiquitous water bodies, rice paddies, and bushland Mixed origin of restocking poultry	[21,39,43,57,68,69]
**Large holding (specifically, commercial layer farms)**	Limited biosecurity, including - Limited control of vehicle movements - Improper management of entry of farm personnel - Failure to impose unauthorized movement - Exchange of farm equipment - Roaming of village chickens around the farm - Unsafe sources of litter/bedding materials Extreme environmental conditions (heat and cold stress, humidity) Vaccination failures	[21,70]
**Live bird markets (LBM)**	Low level of biosecurity, including - Lack of market infrastructure - Inadequate hygienic measures: sanitation and disinfection - No use of personal protection equipment - Lack of awareness about AIV transmission - Keeping chickens and ducks together in the stalls - Exchange of egg-trays and vehicles between LBMs and farms Taking back poultry at the end of market days	[21,27,39,57,59,61,71,72]

**Table 2:** Laboratory-confirmed human cases of high pathogenicity avian influenza (HPAI) H5N1 and low pathogenicity avian influenza (LPAI) H9N2 virus infections in Bangladesh.

AIV Subtypes/ Clade/ Lineage	Year	Patient	Clinical Signs	Poultry Exposure	Case Fatality	References
**HPAI H5N1**						
N/A	2008	15-month-old male	Fever and difficulty in breathing	Exposure to slaughtered chicken	Recovered	[88]
2.2.2	2011	13-month-old female	Fever, cough, and loose stool	Close proximity to well-appearing, sick, or dead birds	Recovered	[82]
2.2.2	2011	31-month-old male	Fever, cough, runny nose, conjunctivitis, vomiting, and diarrhea	Close proximity to backyard poultry, history of visiting live bird market, and handling bird before onset of infection	Recovered	[82]
N/A	2012	26-year-old male	Cough	Exposure to live bird market	Recovered	[89]
N/A	2012	18-year-old male	Cough	Exposure to live bird market	Recovered	[89]
N/A	2012	40-year-old male	Mild illness	Exposure to live bird market	Recovered	[89]
2.3.2.1	2013	23-month-old male	severe pneumonia, meningitis, and disseminated intravascular coagulation	Close proximity to backyard sick chicken	Fatal	[86]
N/A	2015	60-year-old male	Severe acute respiratory signs	Exposure to live backyard poultry	Recovered	[90]
**LPAI H9N2**						
G1	2011	51-month-old female	Fever, headache, runny nose, cough, and sneezing	Close exposure to sick bird	Recovered	[91]
G1	2015	42-month-old female	Mild illness	Close contact with poultry, including sick quails	Recovered	[92]
G1	2015	46-year-old male	Fever	Poultry worker, regular exposure to bird	Recovered	[90]

N/A (Not available): Gene sequences not available in the databases.

**Table 3:** Gene constellation based on the complete genome sequences of 220 HPAI viruses of H5N1 subtype from Bangladesh available in the databases (Global Initiative on Sharing Avian Influenza Data (GISAID) and National Center for Biotechnology Information (NCBI)).

Genotype	Clade 2.2.2	Clade 2.3.4.2	Clade 2.3.2.1c	Clade 2.3.2.1a (Old)	Clade 2.3.2.1a (Old) with H9N2-like *PB1*	Clade 2.3.2.1a (New) R1	Clade 2.3.2.1a (New) R2
**Gene constellation**	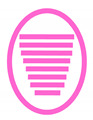	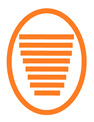	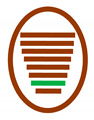	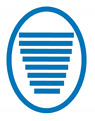	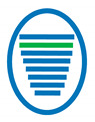	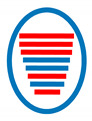	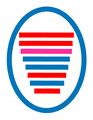
**Features**	All eight gene segments are H5N1 *HA* clade 2.2.2-like.	All gene segments are H5N1 *HA* clade 2.3.4.2-like.	All gene segments except *M*, are H5N1 *HA* clade 2.3.2.1c-like. *M* gene resembling Chinese H9N2 subtype.	All gene segments are H5N1 *HA* clade 2.3.2.1a-like.	All gene segments except *PB1* are H5N1 *HA* clade 2.3.2.1a-like. *PB1* gene resembling Bangladeshi H9N2 viruses under G1-Western lineage.	H5N1 *HA* clade 2.3.2.1a-like *HA*, *NA* and *M* genes. LPAI-like *PB2*, *PB1*, *PA*, *NP*, and *NS* genes. *PA* gene resembling H5N1 clade 2.3.2.1a (new) viruses.	H5N1 *HA* clade 2.3.2.1a-like *HA*, *NA*, and *M* genes. LPAI-like *PB2*, *PB1*, *PA*, *NP*, and *NS* genes. *PA* gene resembling H3N8-like BD LPAI viruses.
**Timeline**	2007–2011	2011	2012	2011–2015	2011–2015	2013–continuing	2017–continuing
**Complete genome sequences available: Year (No.)**	Total: 12 2007 (1), 2010 (8), 2011 (3)	Total: 2 2011 (2)	Total: 2 2012 (2)	Total: 84 2011 (27), 2012 (32), 2013 (4), 2014 (15), 2015 (6)	Total: 8 2011 (2), 2012 (2), 2013 (3), 2015 (1)	Total: 98 2013 (4), 2015 (11), 2016 (25), 2017 (41), 2018 (17)	Total: 14 2017 (9), 2018 (5)

Note: The gene segment order is based on their length from top to bottom, i.e., Polymerase basic 2 (*PB2*), Polymerase basic 1 (*PB1*), Polymerase acidic (*PA*), Hemagglutinin (*HA*), Nucleoprotein (*NP*), Neuraminidase (*NA*), Matrix (*M*), and Nonstructural (*NS*).

## Supplementary Materials

The following are available online at https://www.mdpi.com/1999-4915/12/7/751/s1, **Figure S1:** Phylogenetic relationship of *HA* genes of the Bangladeshi HP H5N1 (a) and LP H9N2 (b) viruses. The generation of trees has been detailed in the legend of Figure 3. **Table S1:** Avian influenza virus *HA* subtypes detected in Bangladesh (based on sequences available in public databases). **Table S2:** Avian influenza virus *NA* subtypes detected in Bangladesh (based on sequences available in public databases).
