# Supplementary material for: Controlling Avian Influenza Virus in Bangladesh: Challenges and Recommendations
Source: Viruses. 2020 Jul 12;12(7):751. doi: 10.3390/v12070751 (PMC7412482; doi:10.3390/v12070751)
Supplement: Supplementary file 1 [file viruses-12-00751-s001.pdf]

viruses

MDPI

Supplementary

Controlling Avian Influenza Virus in Bangladesh: Challenges and Recommendations

Rokshana Parvin [1], Mohammed Nooruzzaman [1], Congriev Kumar Kabiraj [1], Jahan Ara Begum [1], Emdadul Haque Chowdhury [1], Mohammad Rafiqul Islam [1] and Timm Harder [2,*]

[1] Department of Pathology, Faculty of Veterinary Science, Bangladesh Agricultural University, Mymensingh 2202, Bangladesh; rokshana.parvin@bau.edu.bd (R.P.); mohammed.nooruzzaman@bau.edu.bd (M.N.); congriev@bau.edu.bd (C.K.K.); jahan.begum@bau.edu.bd (J.A.B.); emdad001@yahoo.com (E.H.C.); mrislam_bau@yahoo.com (M.R.I.)

[2] National Reference Laboratory for Avian Influenza (NRL-AI), Institute of Diagnostic Virology, Friedrich-Loeffler-Institute, Federal Research Institute for Animal Health, Suedufer 10, 17493 Greifswald-Insel Riems, Germany

* Correspondence: Timm.Harder@fli.de

Abstract: Avian influenza virus (AIV) remains a huge challenge for poultry production with negative repercussions for micro- and macro-economy and public health in Bangladesh. High (HP) H5N1 and low pathogenicity (LP) H9N2 AIV are currently endemic in poultry and both have been reported to infect humans sporadically. Multiple virus introductions of different clades of HPAIV H5N1, reassorted genotypes and on-going diversification of LPAIV H9N2 create a highly volatile virological environment which potentially implicates increased virulence, adaptation to new host species and subsequent zoonotic transmission. Allotropy of poultry rearing systems and supply chains further increase the risk of virus spreading which leads to human exposure and fosters the emergence of new potentially pre-pandemic virus strains. Here we review the epidemiology, focusing on (i) risk factors for virus spreading, (ii) viral genetic evolution, and (iii) options for AIV control in Bangladesh. It is concluded that improved control strategies would profit from the integration of various intervention tools including effective vaccination, enhanced biosecurity practice, and improved awareness of producers and traders, although widespread household poultry rearing significantly interferes with any such strategies. Nevertheless, continuous surveillance associated with rapid diagnosis and thorough virus characterization is the basis of such strategies.

Keywords: Bangladesh; poultry; live bird market; avian influenza virus; geno- and pathotype; control

Table S1. Avian influenza virus HA subtypes detected in Bangladesh (based on sequences available in public databases).

HA subtypes	Subtype combination	Number of isolates/strains	Host	Year of collection
H1	H1N1	4	Domestic duck	2009, 2010
	H1N3	1	Domestic duck	2010
	H1N5	1	Domestic duck	2013
H2	H2N4	4	Domestic duck	2011
	H2N5	1	Domestic duck	2013
H3	H3N1	5	Domestic duck	2017
	H3N2	5	Domestic duck	2010, 2011, 2016, 2017
	H3N6	6	Domestic and wild duck	2010, 2015

	H3N8	7	Domestic duck	2009, 2016, 2018
H4	H4N2	3	Domestic duck	2010, 2017
	H4N6	9	Domestic duck	2008, 2009, 2010, 2015, 2017,2018
H5	H5N1	282	Chicken, domestic duck, quail, turkey, goose, crow, migratory birds, other avian & poultry, human and environment	2007–2018
	H5N2	6	Chicken, domestic duck	2009, 2016, 2018
	H5N3	1	Environment	2017
	H5N6	10	Chicken, domestic and wild duck, wild goose	2017
H6	H6N1	2	Domestic and wild duck	2008, 2015
	H6N2	1	Domestic duck	2016
	H6N7	1	waterfowl	2013
H7	H7N1	5	Domestic and Wild duck	2015, 2016
	H7N2	1	Domestic duck	2016
	H7N5	1	Wild bird (black tailed godwit)	2015
	H7N6	1	Domestic duck	2018
	H7N9	5	Domestic and wild duck, environment	2010, 2012, 2015
H8	-	-	-	-
H9	H9N2	201	Chicken, domestic duck, quail, pigeon, other avian & poultry, environment, and human	2006–2018
H10	H10N1	1	Domestic duck	2014
	H10N6	1	Domestic duck	2015
	H10N7	4	Domestiac duck, chicken	2009
	H10N9	2	Domestic duck	2010
H11	H11N3	9	Domestic duck, environment	2007, 2009, 2010
H12–14	-	-	-	-
H15	H15N9	2	Wild duck	2015
H16	-	-	-	-

Table S2. Avian influenza virus NA subtypes detected in Bangladesh (based on sequences available in public databases).

NA subtypes	Subtype combination	Number of isolates/strains	Host	Year of collection
N1	H1N1	4	Domestic duck	2009, 2010
	H3N1	5	Domestic duck	2017
	H5N1	282	Chicken, domestic duck, quail, turkey, goose, crow, migratory birds, other avian & poultry, human and environment	2007–2018
	H6N1	2	Domestic duck	2008, 2015
	H7N1	5	Domestic and wild duck	2015, 2016
	H10N1	1	Domestic duck	2014
N2	H3N2	5	Domestic duck	2010, 2011, 2016, 2017
	H4N2	3	Domestic duck	2010, 2017
	H5N2	6	Chicken, duck	2009, 2016, 2018
	H6N2	1	Domestic duck	2016
	H7N2	1	Domestic duck	2016
	H9N2	201	Chicken, domestic duck, quail, pigeon, other avian & poultry, human, and environment	2006–2018
N3	H1N3	1	Domestic duck	2010
	H5N3	1	Environment	2017
	H11N3	9	Domestic duck, environment	2007, 2009, 2010
N4	H2N4	4	Domestic duck	2011
N5	H1N5	1	Domestic duck	2013
	H2N5	1	Domestic duck	2013
	H7N5	1	Wild bird (black tailed godwit)	2015
N6	H3N6	6	Domestic and wild duck	2010, 2015
	H4N6	9	Domestic duck	2008, 2009,2010, 2015, 2017, 2018
	H5N6	10	Chicken, domestic and wild duck, wild goose	2017
	H10N6	1	Domestic duck	2015
N7	H6N7	1	Waterfowl	2013
	H10N7	4	Domestic duck	2009
N8	H3N8	6	Domestic duck	2009, 2016, 2018
N9	H7N9	4	Domestic and wild duck, environment	2010, 2012, 2015
	H10N9	2	Domestic duck	2010
	H15N9	2	Wild duck	2015
